# Gap in gender parity: gender disparities in incidence and clinical impact of chronic total occlusion in non-infarct artery in patients with non-ST-segment elevation myocardial infarction and multivessel coronary artery disease

**DOI:** 10.18632/oncotarget.16134

**Published:** 2017-03-11

**Authors:** Mateusz Tajstra, Michał Hawranek, Piotr Desperak, Aneta Ciślak, Mariusz Gąsior

**Affiliations:** ^1^ Third Department of Cardiology, SMDZ in Zabrze, Medical University of Silesia, Katowice, Poland

**Keywords:** gender, sex, chronic total occlusion, non-ST-segment elevation myocardial infarction, percutaneous coronary intervention

## Abstract

A chronic total occlusion in a non-infarct-related artery is an independent predictor of mortality in non-ST elevation myocardial infarction. There are no mortality data about the impact of a chronic total occlusion in patients with non-ST elevation myocardial infarction according to gender. The purpose of this study was to evaluate the prevalence of the chronic total occlusion in in men and women and examine its impact on clinical outcomes. Data from consecutive patients with multivessel coronary artery disease treated in a high-volume center between 2006 and 2012 were included in a prospective registry and divided according to gender and the presence of chronic total occlusion. All of the analyzed patients were followed up for at least 24 months, with all-cause mortality defined as the primary endpoint.

Among the 515 patients who fulfilled the inclusion criteria, 32.8% were female. In the female arm, the 24-month mortality for the groups with and without chronic total occlusion was similar (18.9% and 14.7%, respectively; *p* = 0.47). In contrast, in the male arm, the occurrence of chronic total occlusion was associated with higher 24-month mortality (24.3% vs. 13.4%; *p* = 0.009). Multivariate analysis of the male arm revealed a trend toward a positive association between the occurrence of chronic total occlusion and 24-month mortality (HR 1.62; 95% CI 0.93–2.83; *p* = 0.087). The presence of chronic total occlusion in men is associated with an adverse long-term prognosis, whereas in women this effect was not observed.

## INTRODUCTION

Non-ST-segment elevation myocardial infarction (NSTEMI) remains the most frequently observed presentation of acute coronary syndrome (ACS), and patients with NSTEMI constitute the largest group of patients undergoing percutaneous coronary intervention (PCI) for ACS. Despite significant progress in medical and interventional treatments, the long-term mortality remains high and equal to that of patients with ST-elevation myocardial infarction (STEMI) [1]. The occurrence of chronic total occlusion (CTO) in non infarct-related artery (non-IRA) is present in approximately one-third of patients with NSTEMI and multivessel coronary artery disease (MVCAD), and it is an independent predictor of high mortality [2].

According to work recently published by the European Society of Cardiology (ESC), important differences in diagnoses, medical therapies and invasive treatments between men and women still exist [3].

Considering these facts, as well as the lack of information from published studies on the impact of non-IRA CTO in patients with NSTEMI and MVCAD on mortality by gender, we sought to investigate the prevalence of non-IRA CTO in men and women and to examine its impact on clinical outcomes. Such data may provide significant diagnostic and prognostic information for all medical teams involved in the treatment of patients with NSTEMIs.

## RESULTS

The baseline clinical characteristics of the study groups are presented in Table 1. Of the 515 patients included in the study, 32.8% were female. The prevalence of CTO was similar between men and women. Women with CTO were older, had a higher prevalence of diabetes and obesity and were less frequently current or past smokers than men with CTO. In both arms of the analysis divided according to sex, patients with CTO had a higher rate of previous MI. The in-hospital parameters on admission are listed in Table 2. Women with CTO had lower hemoglobin concentration, higher blood glucose and higher GRACE and CRUSADE risk scores when compared to men with CTO. In both gender arms, significantly lower left ventricle ejection fractions (LVEFs) were recorded in patients with CTO. The angiographic and procedural findings listed in Table 3 differed significantly between the two arms, as well as within both arms between groups with and without CTO. Pharmacotherapy at discharge in studied groups is presented in Table 4. CTO patients in both arms had a higher frequency of 2- and 3-vessel CAD. The in-hospital and long-term outcome analyses are presented in Table 5. In the male arm, the composite end-point rate was higher in the group with CTO (CTO+) compared to the group without (CTO-) after 30 days of follow-up (13.8 *vs*. 7.2, *p* = 0.04). A trend towards a significant difference in the composite end-point rate was also observed during the 24-month follow-up: 37.5% for CTO+ *vs*. 28.9% for CTO- (*p* = 0.09) in the male arm (Figure 2). A significant difference in the all-cause mortality rate in the male arm was observed at the 12-month follow-up, 19.1% for CTO+ *vs*. 8.8% for CTO- (*p* = 0.005), and the difference was sustained at 24 months: 24.3% for CTO+ *vs*. 13.4% for CTO- (*p* = 0.009) (Figure 3). More detailed data about the impact of gender and CTO on individual components of the composite end-point are presented in Table 5 and in Figures 4, 5. In multivariate analysis of the male arm, a trend towards a positive association between the occurrence of CTO and a 24-month mortality was observed (HR 1.62; 95% CI 0.93-2.83; *p* = 0.087) (Table 6, Figures 6, 7).

**Table 1:** Baseline clinical characteristics of the study groups

Variable	Female arm (*N* = 169) (32.8%)			Male arm (*N* = 346) (67.2%)			CTO (+) (*N*=226)
	CTO(+) *N* = 74 (43.8%)	CTO(-) *N* = 95 (56.2%)	*P*	CTO(+) *N* = 152 (43.9%)	CTO(-) *N* = 194 (56.1%)	*P*	Female *vs* Male *P*
Age, years ± SD	71.9 ± 8.1	67.9 ± 10.0	**0.0044**	64.6 ± 9.8	65.0 ± 10.9	0.72	**<0.0001**
Age > 80 years, %	23.0	8.4	**0.0082**	5.3	11.9	**0.033**	**<0.0001**
Arterial hypertension, %	76.7	67.4	0.18	69.3	63.9	0.29	0.25
Prior MI, %	43.8	25.3	**0.011**	48.7	29.9	**0.0004**	0.50
Prior PCI, %	21.9	17.9	0.52	31.3	22.2	0.055	0.14
Atrial fibrillation, %	11.0	4.2	0.092	8.0	5.7	0.39	0.47
Peripheral artery disease, %	20.6	10.5	0.070	16.0	11.3	0.21	0.40
Prior stroke, %	8.2	4.2	0.28	8.7	4.6	0.13	0.91
DM, %	46.6	41.0	0.47	32.7	26.8	0.24	0.44
DM insulin treatment, %	32.9	27.4	0.44	12.0	9.3	0.41	**0.0002**
Hypercholesterolemia, %	37.0	33.7	0.66	30.0	23.7	0.19	0.29
Obesity, %	41.1	35.8	0.48	20.7	25.3	0.32	**0.0013**
COPD, %	5.3	1.4	0.18	7.3	4.1	0.20	0.64
History of cigarette smoking, %	23.3	30.5	0.30	44.0	44.9	0.88	**0.0027**
Current cigarette smoker, %	9.6	18.9	0.091	20.7	24.2	0.43	**0.039**
Familial history of MI, %	23.3	26.3	0.65	18.7	22.2	0.43	0.42

COPD = chronic obstructive pulmonary disease; DM = diabetes mellitus; MI = myocardial infarction; PCI = percutaneous coronary intervention; SD = standard deviation.

**Figure 1:**
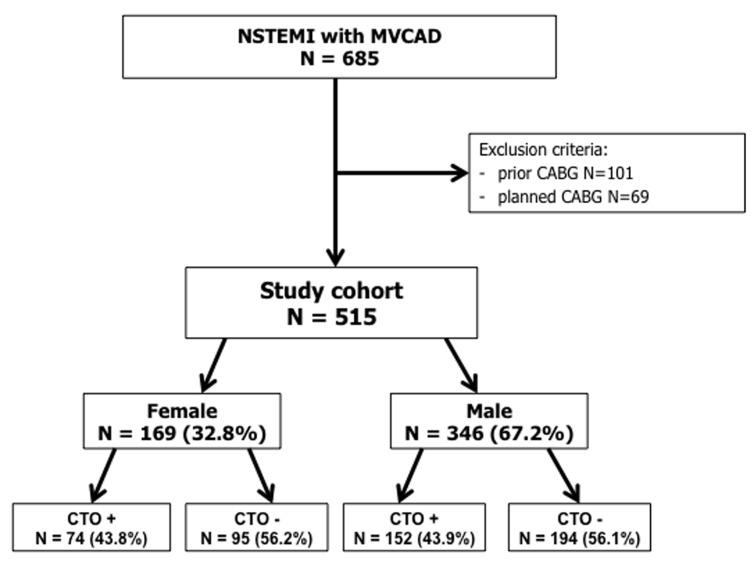
Study flow chart

**Table 2:** In-hospital data on admission of study groups.

Variable	Female arm (*N* = 169) (32.8%)			Male arm (*N* = 346) (67.2%)			CTO (+) (*N* = 226)
	CTO(+) *N* = 74 (43.8%)	CTO(-) *N* = 95 (56.2%)	*P*	CTO(+) *N* = 152 (43.9%)	CTO(-) *N* = 194 (56.1%)	*P*	Female *vs* Male *P*
Chest pain, %	95.9	89.5	0.12	92.0	93.8	0.51	0.27
Killip class III, %	8.1	7.4	0.86	6.6	2.6	0.07	0.68
Killip class IV, %	4.0	2.1	0.46	0.7	1.0	0.71	0.070
Heart rate, bpm ± SD	82 ± 22	81 ± 15	0.75	82 ± 17	76 ± 15	**0.0005**	0.89
SBP, mmHg ± SD	148 ± 34	152 ± 29	0.39	148 ± 29	151 ± 29	0.44	0.95
DBP, mmHg ± SD	85 ± 21	86 ± 19	0.66	90 ± 17	89 ± 18	0.76	0.077
BMI, kg/m^2^ (Q1-Q3)	29 (24-31)	29 (26-31)	0.69	27 (25-30)	28 (25-30)	0.96	0.50
WBC, tys/uL (Q1-Q3)	10 (8-13)	10 (8-12)	0.87	10 (8-12	9 (7-11)	**0.01**	0.39
Hemoglobin, mmol/L ± SD	8.0 ± 0.9	7.9 ± 1.1	0.28	8.8 ± 1.1	8.7 ± 1.0	0.44	**<0.0001**
Glucose, mmol/L (Q1-Q3)	7.4 (5.9-12.4)	7.9 (6.1-10.8)	0.23	6.6 (5.6-8.3)	6.2 (5.5-7.7)	0.20	**0.020**
Serum creatinine, umol/L (Q1-Q3)	83 (67-112)	74 (59-106)	0.83	87 (75-105)	83 (70-103)	0.88	0.40
GFR, ml/min/1.73 m^2^ (Q1-Q3)	63 (44-84)	74 (46-96)	**0.04**	81 (65-96)	82 (65-104)	0.30	**<0.0001**
GRACE risk score, points (Q1-Q3)	133 (112-155)	119 (104-143)	**0.004**	115 (98-136)	112 (90-130)	0.12	**<0.0001**
CRUSADE risk score, points (Q1-Q3)	41 (32-51)	34 (26-49)	0.17	22 (15-34)	20 (14-29)	0.08	**<0.0001**
LVEF, % (Q1-Q3)	42 (35-48)	45 (40-50)	**0.02**	40 (30-48)	46 (38-50)	**<0.0001**	0.16
LVEF ≤ 35%, %	25.7	14.8	0.08	38.2	17.9	**0.0004**	0.071

BMI = body mass index; DBP = diastolic blood pressure; GFR = glomerular filtration rate; GRACE = Global Registry of Acute Coronary Events; LBBB = left bundle branch block; LVEF = left ventricular ejection fraction; RBBB = right bundle branch block; SBP = systolic blood pressure; Q1-Q3 = quartile 1 and quartile 3; SD = standard deviation; WBC = white blood cells.

**Table 3:** Angiographic and procedural characteristics of study groups.

Variable	Female arm (*N* = 169) (32.8%)			Male arm (*N* = 346) (67.2%)			CTO (+) (*N* = 226)
	CTO(+) *N* = 74 (43.8%)	CTO(-) *N* = 95 (56.2%)	*P*	CTO (+) *N* = 152 (43.9%)	CTO (-) *N* = 194 (56.1%)	*P*	Female vs Male *P*
Femoral access, %	93.2	93.7	0.91	94.1	94.8	0.76	0.81
Radial access, %	6.8	6.3	0.91	5.9	5.2	0.76	0.96
2-vessel CAD, %	66.3	45.9	**0.008**	73.7	39.5	**<0.0001**	0.35
3-vessel CAD, %	54.1	33.7	**0.008**	60.5	26.3	**<0.0001**	0.35
LM CAD, %	14.9	8.4	0.19	9.2	9.3	0.98	0.20
PCI IRA, %	83.8	93.7	**0.04**	90.1	94.3	0.14	0.17
PCI ad hoc, %	91.9	96.6	0.21	93.4	95.6	0.39	0.70
LM, %	6.4	2.2	0.19	3.6	2.7	0.64	0.38
LAD, %	32.3	31.5	0.92	38.0	36.1	0.73	0.44
LCx, %	30.6	34.8	0.59	31.4	35.0	0.50	0.92
RCA, %	30.6	31.5	0.92	27.0	26.2	0.88	0.60
Restenotic lesion, %	6.5	6.7	0.94	8.8	4.9	0.17	0.58
Bifurcation of LM	4.8	3.4	0.65	3.6	4.4	0.75	0.69
Bifurcation other than LM	11.3	14.6	0.55	22.6	15.3	0.09	0.06
Baseline TIMI flow grade 0–1, %	17.7	21.3	0.58	24.1	30.6	0.20	0.32
Stent placement, %	91.9	95.5	0.36	92.0	93.4	0.61	0.99
Balloon predilatation, %	66.1	68.5	0.75	65.0	68.3	0.53	0.87
Balloon postdilatation, %	11.3	7.9	0.48	10.9	12.0	0.77	0.94
DES, %	17.7	9.0	0.11	16.1	16.4	0.94	0.77
Procedural glycoprotein IIb/IIIa inhibitor, %	4.8	6.7	0.63	14.6	9.3	0.14	**0.05**
Dissection, %	6.5	6.7	0.94	9.5	5.5	0.17	0.48
No/slow reflow, %	0.0	1.1	0.40	2.2	1.1	0.43	0.24
Final TIMI flow grade 3 after PCI, %	95.2	97.8	0.38	89.8	97.3	**0.005**	0.21
Procedural success of PCI IRA, %	95.2	96.6	0.65	87.6	95.6	**0.008**	0.09
PCI of additional artery during hospitalization, %	13.5	36.8	**0.0007**	18.4	34.5	**0.0009**	0.35
Angiographic success of all lesions, %	91.9	95.5	0.36	86.9	92.9	0.07	0.30

CAD = coronary artery disease; LCx = left circumflex artery; DES = drug eluting stent; GP = glycoprotein; IRA = infarct-related artery; LAD = left anterior descending artery; LM = left main; PCI = percutaneous coronary intervention; RCA = right coronary artery; TIMI = Thrombolysis in Myocardial Infarction.

**Table 4:** Pharmacotherapy at discharge.

Variable	Female-arm (*N* = 169) (32.8%)			Male-arm (*N* = 346) (67.2%)			CTO (+) (*N*=226)
	CTO (+) *N* = 74 (43.8%)	CTO (-) *N* = 95 (56.2%)	*P*	CTO (+) *N* = 152 (43.9%)	CTO (-) *N* = 194 (56.1%)	*P*	Female vs Male-arm
Acetylsalicylic acid, %	100.0	97.4	0.22	100.0	99.4	0.39	0.99
P2Y12 inhibitors, %	98.2	93.6	0.19	90.6	93.2	0.40	0.060
Angiotensin-converting-enzyme inhibitors/ Angiotensin receptor blockers, %	91.2	88.5	0.60	89.8	90.3	0.87	0.42
Beta-adrenergic antagonists, %	96.5	96.2	0.92	96.9	95.5	0.54	0.90
Loop diuretics, %	38.6	20.5	0.021	32.3	17.6	0.0031	0.40
Aldosterone antagonist, %	47.4	35.9	0.18	40.9	28.4	0.023	0.76
Oral anticoagulants, %	8.8	2.6	0.11	3.2	1.1	0.21	0.10
Nitrates, %	40.3	32.0	0.32	32.3	29.6	0.61	0.29
Statins, %	96.5	94.9	0.65	93.7	96.0	0.36	0.44

**Table 5:** In-hospital, early and long-term outcomes of study groups

Variable	Female arm (*N* = 169) (32.8%)			Male arm (*N* = 346) (67.2%)			CTO (+) (*N*=226)
	CTO(+) *N* =74 (43.8%)	CTO(-) *N* =95 (56.2%)	*P*	CTO(+) *N* = 152 (43.9%)	CTO(-) *N* = 194 (56.1%)	*P*	Female vs Male *P*
**In-hospital outcomes**							
Death, %	6.8	3.2	0.27	5.3	2.6	0.19	0.65
Non-fatal MI, %	2.7	2.1	0.80	1.3	2.1	0.60	0.46
TVR, %	2.7	2.1	0.80	3.9	2.1	0.30	0.63
Bleeding, %	4.0	7.4	0.36	2.0	2.6	0.71	0.36
Stroke, %	0.0	1.0	0.38	0.7	0.0	0.26	0.48
Cardiogenic shock, %	6.8	4.2	0.46	3.9	1.6	0.16	0.36
Hospital stay, days (Q1-Q3)	8 (5-10)	7 (4-9)	0.96	6 (4-8)	5 (4-7)	**0.03**	**0.02**
**Post-discharge outcomes**							
**30-days** MACE, %	16.2	8.4	0.12	13.8	7.2	**0.04**	0.63
Death, %	8.1	3.2	0.16	8.5	4.1	0.09	0.91
Non-fatal MI, %	5.4	4.2	0.72	3.9	3.1	0.67	0.62
Revascularization in ACS, %	4.0	4.2	0.96	5.3	3.1	0.31	0.69
**12-month** MACE, %	33.8	27.4	0.37	29.6	21.1	**0.07**	0.52
Death, %	16.2	11.6	0.38	19.1	8.8	**0.005**	0.60
Non-fatal MI, %	14.9	15.8	0.87	9.9	11.9	0.56	0.27
Revascularization in ACS, %	12.2	13.7	0.77	9.9	10.8	0.77	0.60
**24-month** MACE, %	40.5	32.6	0.29	37.5	28.9	0.09	0.66
Death, %	18.9	14.7	0.47	24.3	13.4	**0.009**	0.36
Non-fatal MI, %	20.3	16.8	0.57	13.2	13.4	0.94	0.16
Revascularization in ACS, %	13.5	15.8	0.68	12.5	12.9	0.91	0.83

ACS = acute coronary syndrome; MACE = major adverse cardiac events; MI = myocardial infarction; Q1-Q3 = quartile 1 and quartile 3; TVR = target vessel revascularization.

**Figure 2:**
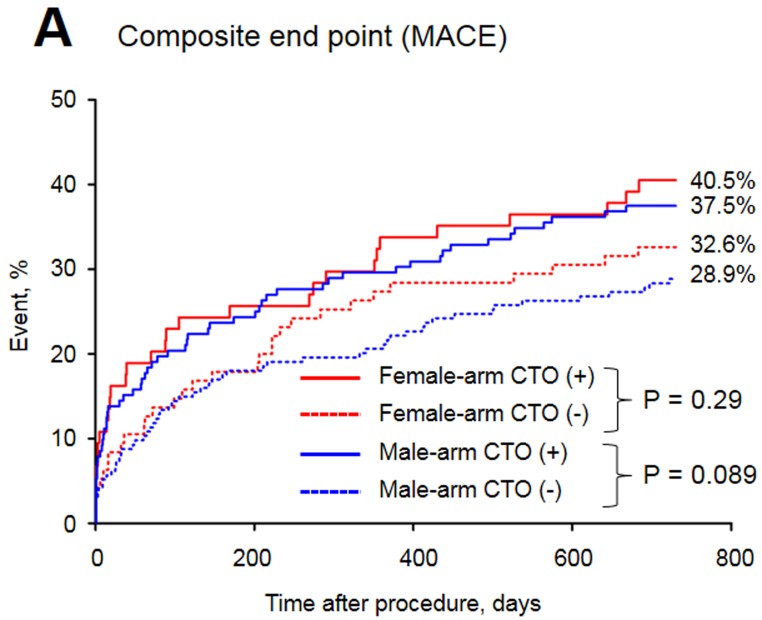
Major adverse cardiac events in the study groups

**Figure 3:**
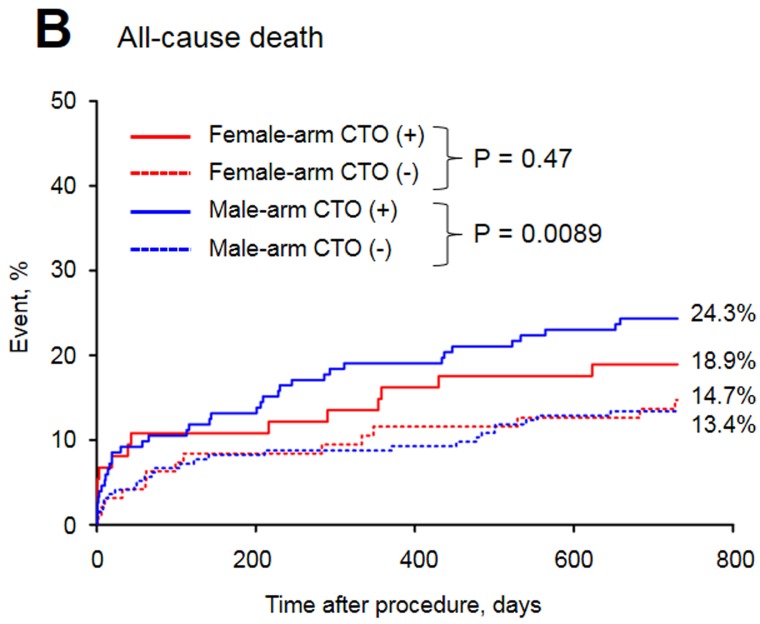
Long-term mortality of the study groups

**Figure 4:**
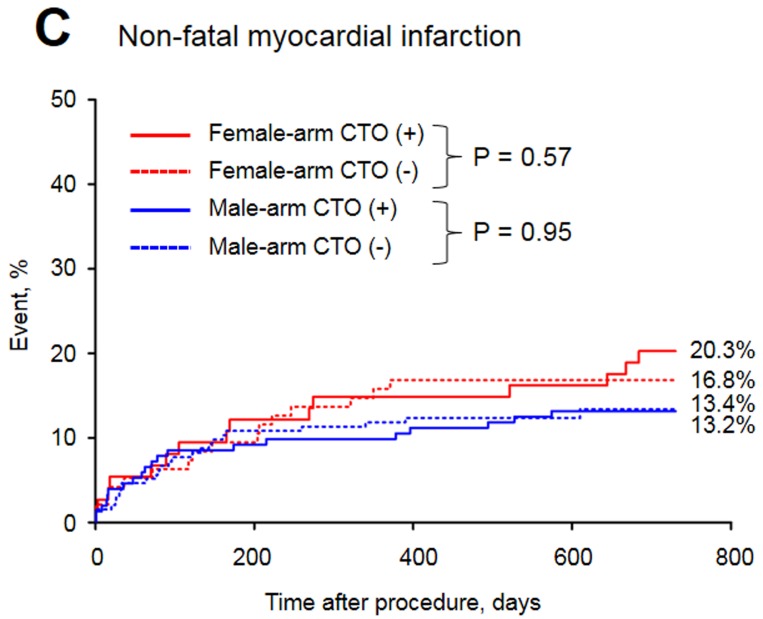
Long-term myocardial infarction rate in the study groups

**Figure 5:**
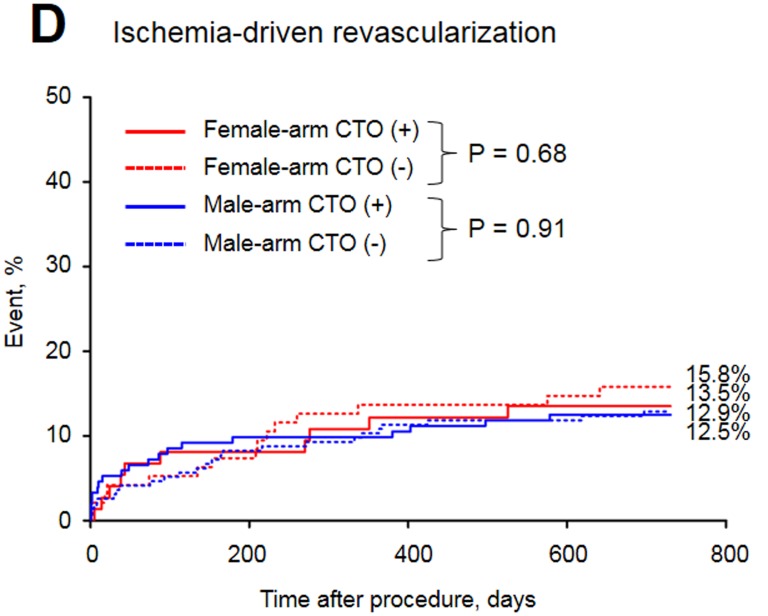
Long-term revascularization rate in the study groups

**Table 6:** Long-term clinical events

Female arm, CTO (+)	unadjusted HR	95% CI	*P*	adjusted HR	95% CI	*P*
24-month MACE, %	1.33	0.80 – 2.19	0.27	-	-	-
Death, %	1.34	0.64 – 2.81	0.44	-	-	-
Non-fatal MI, %	1.21	0.60 – 2.45	0.59	-	-	-
ACS-driven revascularization, %	0.85	0.38 – 1.89	0.69	-	-	-
**Male arm, CTO (+)**	**unadjusted HR**	**95% CI**	***P***	**adjusted HR**	**95% CI**	***P***
24-month MACE, %	1.41	0.97 – 2.03	0.070	-	-	-
Death, %	1.94	1.17 – 3.20	0.0098	1.62	0.93 – 2.83	0.087
Non-fatal MI, %	0.98	0.55 – 1.76	0.96	-	-	-
ACS-driven revascularization, %	0.98	0.54 – 1.78	0.96	-	-	-

Unadjusted and adjusted hazard ratios in multivariate analysis.

ACS = acute coronary syndrome; CI = confidence interval; CTO = chronic total occlusion; HR = hazard ratio; MACE = major adverse coronary events; MI = myocardial infarction;

**Figure 6:**
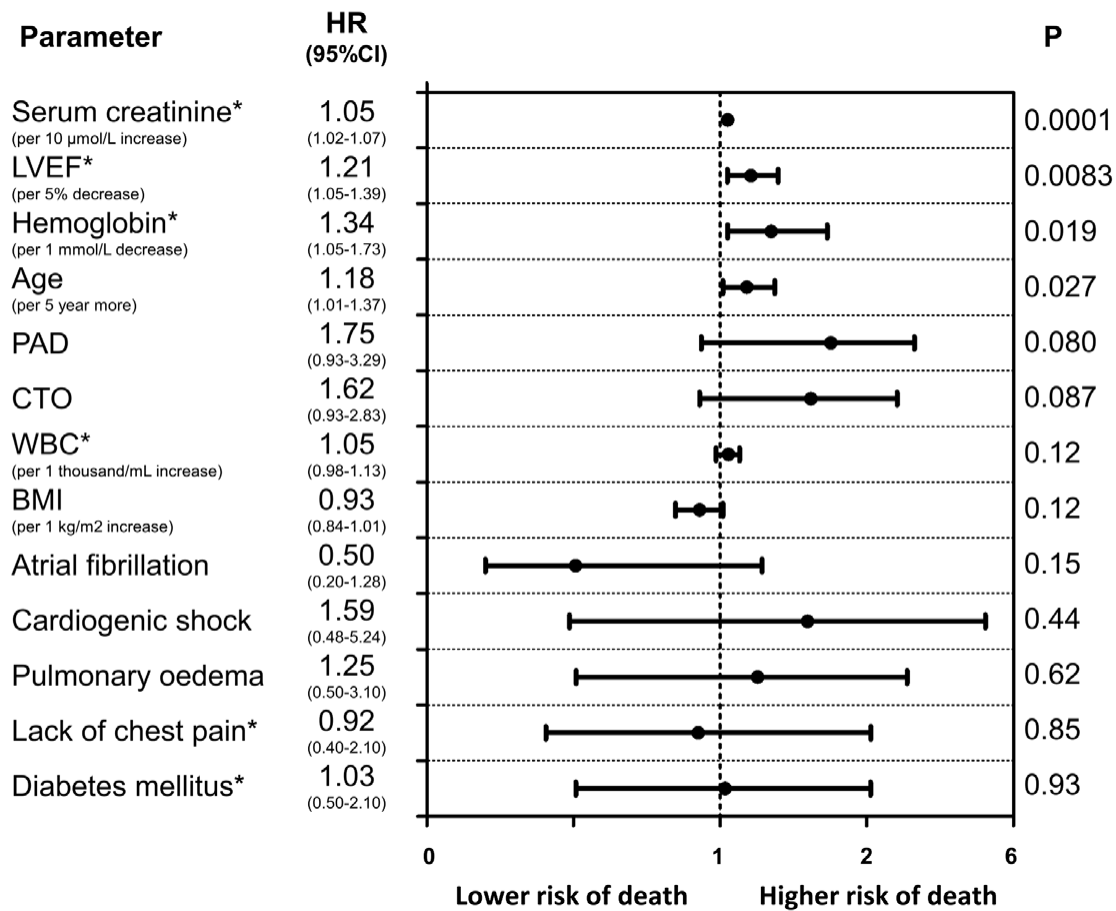
Predictors of long-term mortality (Cox proportional hazards model results) in men Variables are shown in descending order of Wald X^2^ values.

**Figure 7:**
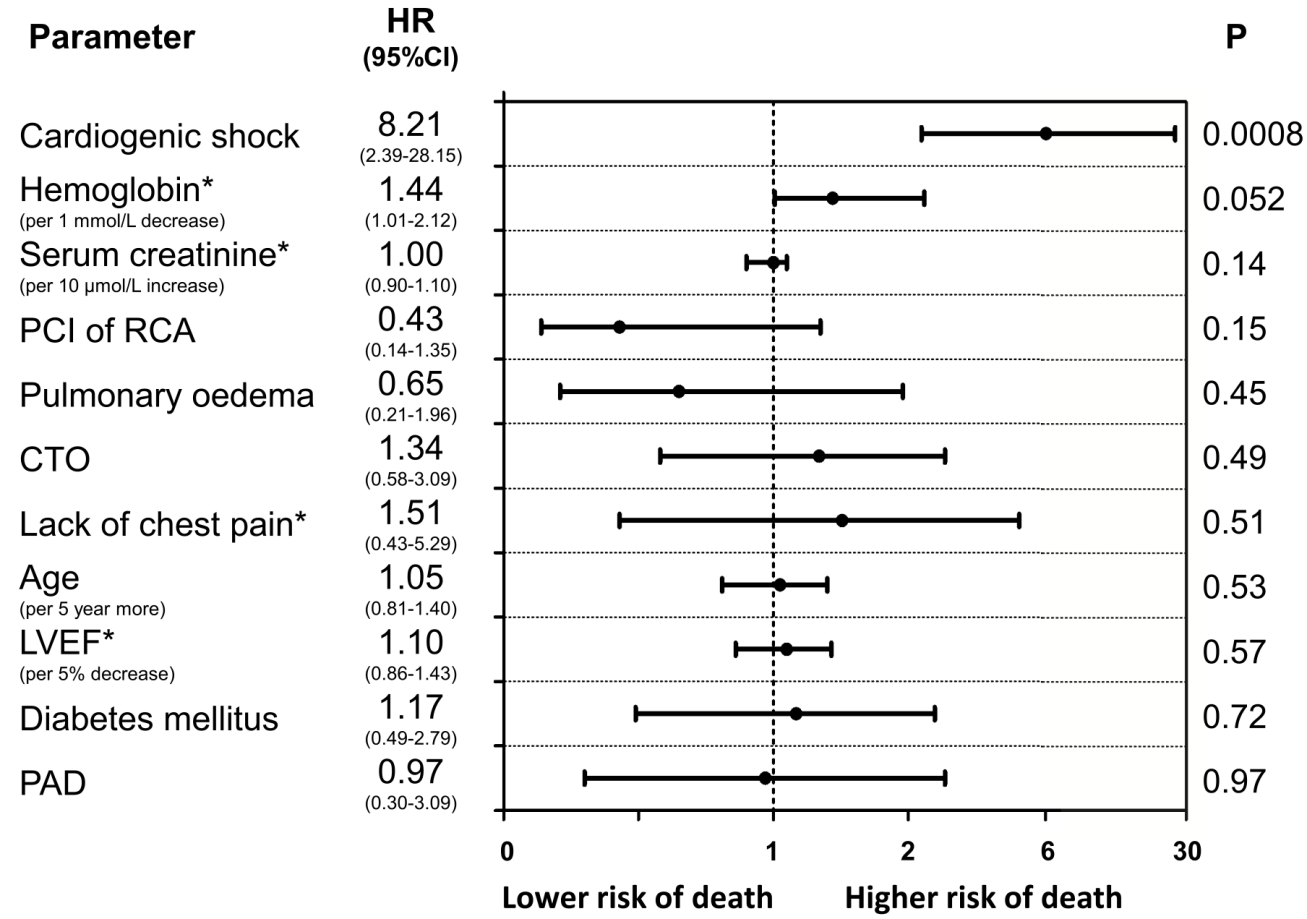
Predictors of long-term mortality (Cox proportional hazards model results) in women Variables are shown in descending order of Wald X^2^ values.

## DISCUSSION

Gender has been associated with discrepant management of NSTE-ACS. Compared with men, women presenting with NSTEMI are up to 30% less likely to be referred for cardiac testing and catheterization and receive novel therapies less frequently [5-7]. Women, have a two-fold higher risk of in-hospital death. However, some studies have reported no difference in the 12-month mortality between men and women using both unadjusted and adjusted discharge medications by propensity score [8-10]. Therefore, the impact of gender on clinical outcomes in patients with NSTE-ACS remains controversial. In light of these facts, potential interactions between gender and non-IRA CTO should be evaluated.

The purpose of the present investigation was to evaluate the incidence of non-IRA CTO in men and women and to examine its impact on clinical outcomes. In this study involving unselected, consecutive NSTEMI patients with MVCAD treated in a high-volume center, we found essential clinical implications which can be summarized as follows: first, CTO was very frequent in this cohort of patients, and there was a similar prevalence in men and women (nearly 44%); second, despite the fact that women presented a higher risk profile compared to men in the subpopulation with non-IRA CTO, no difference in early and long-term mortality according to gender was observed; third, the presence of non-IRA CTOs in the male group was associated with an adverse long-term prognosis, whereas in female patients this effect was not observed; and finally, multivariate analysis revealed a trend towards a positive association between the occurrence of CTO and 24-month mortality only in men.

In this study, over 40% of the patients had non-IRA CTO. This could be partially explained by the fact that our center is a referral center, and we treat the most challenging cases. A significant number of patients were aged above 80 years, had prior coronary revascularization and more severely impaired ejection fraction and were in Killip class III and IV. All of the abovementioned factors contributed to a high risk of advanced and complex coronary artery disease. Additionally, our findings of a very high percentage of CTO was consistent with previous studies in similar populations of patients [2].

There were noticeable differences in baseline and angiographic characteristics between women and men overall and in the non-IRA CTO cohort. Possible reasons for this may be that women generally present with ischemic coronary symptoms later than men, presumably because of the protective effects of estrogen, which prevents coronary atherosclerosis until menopause [11]; women therefore tend to have greater comorbidity and less favorable angiographic characteristics, including smaller caliber of vessels and more disseminated disease.

Interestingly, this analysis showed that the presence of non-IRA CTO was associated with an adverse long-term prognosis only among men; multivariate analysis revealed a trend towards a positive association between the occurrence of CTO and 24-month mortality only in men. One possible explanation may be the limited sample size of the female cohort in the present study. Additionally, this finding seems to be consistent with analyses showing a survival benefit after successful CTO PCI in male patients only [12]. However, in the recently published results of the EXPLORE (Evaluating Xience V and Left Ventricular Function in Percutaneous Coronary Intervention on Occlusions after ST-Elevation Myocardial Infarction) trial, there were no differences in primary outcomes according to the gender subgroup analysis [13].

### Limitations

This study is a single-center observational study derived from a real-life practice with inherent weaknesses related to retrospective analysis. The results of our multivariate analysis may be biased due to the potential impact of important factors that are not available in our database. All mortality data obtained from the National Health Fund were of all-cause. We had no insight into the cause of death (i.e., cancer- or cardiovascular-caused death).

## CONCLUSIONS

The presence of CTO in men is associated with an adverse long-term prognosis, whereas in women this effect was not observed. This study significantly adds to the existing literature investigating the clinical importance of CTO in patients with non-ST elevation myocardial infarction and differences related to gender.

## MATERIALS AND METHODS

### Study population

From January 2006 to December 2012 a total of 1242 patients with NSTEMI were treated in a high-volume PCI center. In this cohort of patients, 685 who were referred for urgent coronary angiography had MVCAD. Patients who qualified for coronary artery bypass graft or had a prior history were excluded from the study. A total of 515 patients were enrolled in this study (Figure 1). The patients were divided according to gender and subsequently divided with regard to the presence of CTO. CTO of the main coronary arteries other than IRA were found in both arms: 74 (43.8%) women (female arm, CTO+ group) and 152 (43.9%) men (male arm, CTO+ group). All of the included patients underwent coronary angiography with PCI using standard devices.

All interventional and therapeutic strategies, including the choice of stent type and periprocedural anti-thrombin and antiplatelet therapy, were made at the discretion of the operator or heart team. Before and after the intervention, pharmacological treatment was administered according to the current ESC guidelines [3].

The demographic, clinical and angiographic data collected during the current hospitalization were retrieved from the prospectively recorded Silesian Center for Heart Disease Electronic Database. The angiographic parameters were recorded on the basis of a visual assessment by two experienced interventional cardiologists. The follow-up data with exact dates of death, MI and ACS driven revascularization were obtained from the official National Health Fund records. Follow-up status at 24 months was available for 100% of all of the included patients. The study protocol was approved by the local ethics committee, and informed consent was obtained from all patients.

### Definitions

NSTEMI was defined as the absence of ST segment elevation of ≥2 mm in contiguous chest leads, ST segment elevation of ≥1 mm in 2 or more standard leads, or new left bundle branch block and the presence of positive cardiac necrosis markers [3,4]. The presence of MVCAD was defined as significant stenosis of one or more major epicardial coronary arteries remote from the IRA as determined by visual assessment involving the territory of the right, left anterior descending (LAD) or the left circumflex coronary vessels. Stenosis ≥50% of the diameter in the left main or proximal segment of the LAD and stenosis ≥70% of the diameter in other segments were considered hemodynamically significant. A coronary artery was considered to be an IRA (culprit) if one of the following criteria was present: definite or suspected thrombus, ruptured or ulcerated plaque, presence of thrombolysis in myocardial infarction (TIMI) flow grade ≤2, and tight stenosis ≥70% consistent with non-invasive ischemia tests. A CTO was defined as a non-IRA with 100% luminal narrowing before PCI and without anterograde flow or with anterograde or retrograde filling through collateral vessels. The differentiation between a CTO and acute occlusion was based on a combination of the following factors: morphology of the occlusion (presence of fresh thrombus, bridge, and ipsi- or contra-lateral collaterals), the ECG recording, echocardiogram findings, and a possible history of prior documented acute coronary events in the same territory.

The composite end-point of major adverse coronary events (MACE) was defined as the following: all-cause mortality, non-fatal MI and ACS-driven revascularization over a long-term observation period. Non-fatal MI was defined as an ischemic event that met the European Society of Cardiology/American College of Cardiology criteria for myocardial infarction and was clinically separate from the baseline ACS at the time of admission [4]. Periprocedural non-fatal MI was defined as an event that occurs in first 24 hours after index PCI, also consistent with the expert consensus document of the Universal Definition of MI (clinical manifestation and >20% change between first and second sample of myocardial necrosis markers after PCI) [4]. ACS-driven revascularization was defined as an additional, unplanned PCI or coronary artery bypass grafting of a previously revascularized coronary artery that was performed as an urgent procedure due to ACS.

### Statistical analyses

Comparisons between CTO+ and CTO- groups were stratified by gender. The statistical analysis included comparison of baseline angiographic and procedural characteristics and the in-hospital and 30-day, 12-month and 24-month adverse events. The analyzed variables are expressed as numbers and percentages. The continuous variables were summarized using an arithmetic mean with standard deviation (SD) for data following a normal distribution, or a median with an interquartile range (IQR) for data with a non-normal distribution. Categorical variables are presented as percentages. The 12-month composite end-point, combining all-cause mortality, non-fatal MI and ACS-driven revascularization, was analyzed using the Kaplan-Meier method for all patients. Multivariate logistic regression analysis was performed in both men and women. Candidate variables for adjusted analysis were: 3-vessel CAD, absence of chest pain on admission, age, atrial fibrillation, body mass index, cardiogenic shock during hospitalization, chronic obstructive pulmonary disease, chronic total occlusion of non-culprit vessel, creatinine kinase Muscle-Brain type on admission, diastolic blood pressure on admission, glucose on admission, hemoglobin on admission, heart rate on admission, diabetes mellitus on insulin treatment, left bundle branch block, left main coronary artery disease, left ventricular ejection fraction, percutaneous coronary intervention of right coronary artery, peripheral artery disease, prior myocardial infarction, serum creatinine on admission, pulmonary edema, success of percutaneous coronary intervention in culprit vessel, systolic blood pressure on admission, ST deviations on admission, and white blood count on admission. A two-sided p-value ≤ 0.05 was considered significant. The STATISTICA 10 software (StarSoft Inc., Tulsa, Oklahoma), MedCalc (MedCalc Software, Mariakerke, Belgium) and SPSS ver. 17.0.1 (SPSS, Inc., Chicago, Illinois) were used for the calculations.
